# Endoscopic ultrasound-guided perforator-targeted embolization for gastric variceal bleeding due to left-sided portal hypertension

**DOI:** 10.1055/a-2776-5431

**Published:** 2026-01-28

**Authors:** Zhihong Wang, Xuecan Mei, Yuchuan Bai, Qianqian Zhang, Zhuang Zeng, Derun Kong

**Affiliations:** 1 36639 Department of Gastroenterology, The First Affiliated Hospital of Anhui Medical University, Key Laboratory of Digestive Diseases of Anhui Province, Hefei, China

Left-sided portal hypertension (LSPH), an uncommon segmental variant comprising under 5% of all portal hypertension cases 1 , is typically due to splenic vein thrombosis or occlusion resulting from chronic pancreatitis. Bleeding from gastric varices (GVs) in this setting is challenging to manage. Surgical splenectomy or splenic artery embolization remains the traditional standard 2 , but these procedures are invasive and often unsuitable for high-risk patients. Conventional endoscopic cyanoacrylate injection has limited efficacy because the feeding perforating vessels are usually located deep within the gastric wall.

**Fig. 1:**
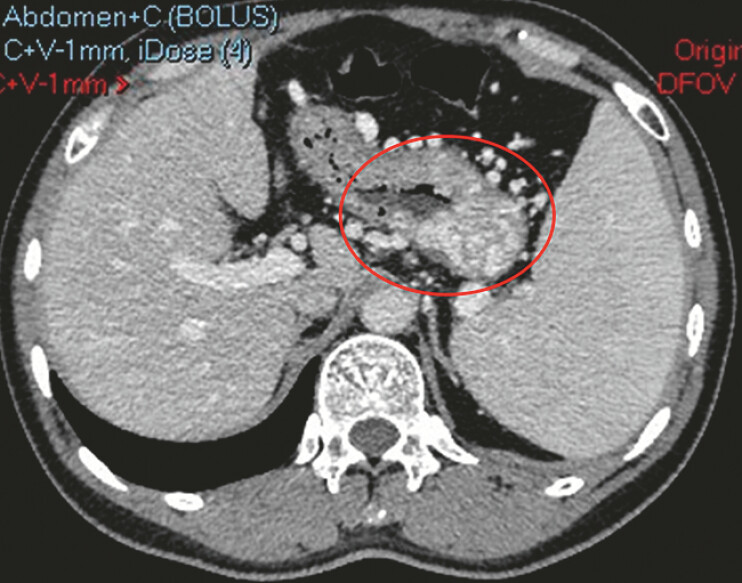
Abdominal computed tomographic angiography shows multiple dilated and tortuous vascular shadows in the stomach.

**Fig. 2:**
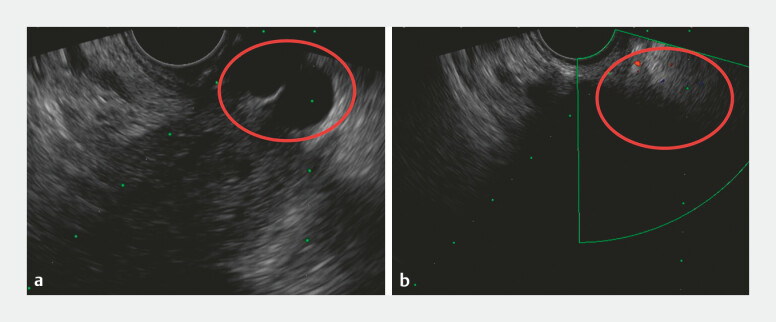
**a** Endoscopic ultrasonography (EUS) reveals collateral vessels of gastric varices at the fundus. **b** Doppler ultrasonography demonstrates the absence of the blood flow signal.

**Fig. 3:**
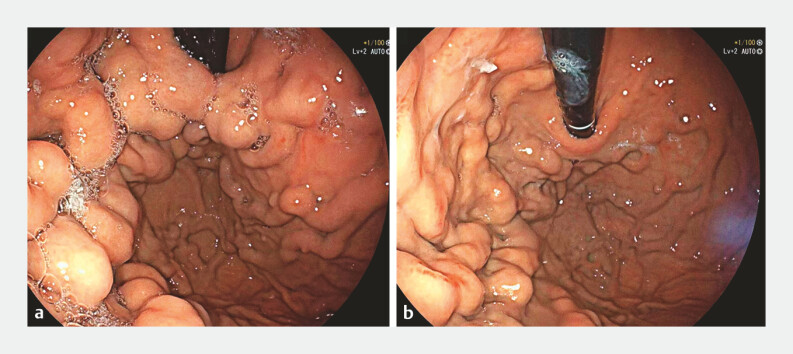
**a** Clustered gastric varices are visible before treatment. **b** Marked regression of the varices is observed post-treatment.

A 43-year-old man with recurrent pancreatitis presented with melena. Contrast-enhanced computed tomography showed splenic vein narrowing and multiple gastric fundal collaterals, consistent with LSPH ( Fig. 1 ). The patient declined surgery and underwent EUS-guided vascular intervention. EUS revealed multiple anechoic channels in the fundus and a 0.8-cm perforating vessel connecting the extramural and submucosal varices ( Fig. 2 **a** ). Under real-time Doppler guidance, stepwise embolization of the perforating vessel was performed using 3 mL of N-butyl-2-cyanoacrylate combined with 10 mL of polidocanol, resulting in the complete disappearance of flow ( Fig. 2 **b** ). Follow-up endoscopy confirmed hemostasis without recurrent bleeding ( Fig. 3 **a, b** , Video 1 ).

EUS-guided targeted embolization of a perforating vessel in left-sided portal hypertension. Doppler imaging identifies the perforating vessel feeding gastric varices. Stepwise injection of cyanoacrylate and polidocanol leads to complete flow cessation, confirmed by real-time EUS and post-procedure endoscopy. EUS, endoscopic ultrasonography.Video 1

Compared with previously reported techniques, our approach represents a conceptual advancement 3 4 . We targeted the perforating vessel, the hemodynamic bridge between extramural and submucosal varices, rather than the variceal sac itself. This perforator-focused embolization allows precise obliteration of the inflow channel with a smaller glue volume, enhanced safety, and potentially more durable hemostasis. The adjunctive use of polidocanol provides additional sclerosant reinforcement and reduces the risk of distal embolization.

To our knowledge, this is among the first detailed video demonstrations of EUS-guided perforator-targeted embolization for LSPH-related GV bleeding, offering a minimally invasive, surgery-sparing treatment option.

Endoscopy_UCTN_Code_TTT_1AS_2AG
